# Integrated Profiling of TNF Superfamily Ligands and Lung Inflammatory Markers in Tuberculosis-Type 2 Diabetes Mellitus Comorbidity

**DOI:** 10.3390/medsci14060613

**Published:** 2026-09-28

**Authors:** Nandita Ganesh, Rajesh Kumar Radhakrishnan, Fathima Sumaira, Jai Priya Govindasamy Venugopal, Bhavani Perumal Kannabiran, Uma Devi Ranganathan, Syed Hissar, Ramakrishna Vankayalapati, Nathella Pavan Kumar

**Affiliations:** 1 ICMR National Institute for Research in Tuberculosis, No. 1, Mayor Sathyamoorthy Road, Chetpet, Chennai 600031, Tamil Nadu, India; gnandita2020@gmail.com (N.G.);; 2 Division of Infectious Diseases, Allergy and Immunology, School of Medicine, Saint Louis University, St. Louis, MO 63104, USA; 3 Department of Biotechnology, ACTech Campus, Anna University, Kotturpuram, Chennai 600025, Tamil Nadu, India; sumairashakir12@gmail.com; 4 RePORT International CRDF Global Funded Extramural Project Hosted at ICMR National Institute for Research in Tuberculosis, No. 1, Mayor Sathyamoorthy Road, Chetpet, Chennai 600031, Tamil Nadu, India

**Keywords:** tuberculosis, diabetes mellitus, TB-T2DM comorbidity, acute-phase proteins, TNF superfamily signaling, lung markers

## Abstract

**Background:** Type 2 diabetes mellitus (T2DM) increases susceptibility to tuberculosis (TB) and contributes to immune dysregulation, exaggerated inflammation and poor clinical outcomes. Acute-phase proteins (APPs), lung-associated markers (LMs) and TNF superfamily ligands play a crucial role in inflammatory and immune signaling; however, an integrated approach in understanding the inflammatory profile and their immunological association in TB-T2DM remains underexplored. **Methods:** Plasma levels of APPs and LMs including CRP, SAA1, Haptoglobin, α2M, IDO, CHI3L1, S100A8, S100A9 and granulysin were evaluated for the study cohort of 176 individuals (TB = 44, TB–T2DM = 44, T2DM-only = 44 and healthy-controls = 44). TNF superfamily ligands, including 4-1BBL, APRIL, BAFF, CD30L, CD40L, EDA-1, Fas-L, GITRL, LIGHT, RANKL, NGF- β, TNF-β, TRAIL and TWEAK, were measured using a multiplex immunoassay for 88 individuals (TB = 22, TB–T2DM = 22, T2DM-only = 22 and healthy-controls = 22). Spearman correlation analysis, protein–protein interaction (PPI) network analysis and pathway enrichment analysis were also performed. **Results:** TB-T2DM individuals exhibited significantly increased inflammatory burden compared with TB, T2DM and HC groups. Increased levels of CRP, SAA1, S100A8, S100A9, haptoglobin, CH3IL1 and α2M were observed (*p* < 0.05), indicating amplified systemic and pulmonary inflammation. TNF superfamily ligands including BAFF, APRIL, CD40L, GITRL, LIGHT and TRAIL were significantly altered in TB-T2DM individuals, suggesting dysregulated TNF-mediated immune signaling. Correlation analysis revealed strong associations between HbA1c levels and inflammatory markers including BAFF, TRAIL, GITRL, LIGHT, CD40L, APRIL, S100A8 and SAA1 (*p* < 0.05). Network and enrichment analyses identified significant involvement of cytokine–cytokine receptor interaction, IL-17 signaling, NF-κB signaling, apoptosis and necroptosis pathways. **Conclusions:** Overall, TB-T2DM comorbidity is characterized by exaggerated inflammatory responses and altered TNF superfamily ligand signaling associated with poor glycemic control. This interconnected immune dysregulation may provide future insights into biomarkers and pathway-related investigation to establish more clinical and biological relevance.

## 1. Introduction

Type 2 diabetes mellitus (T2DM) is a well-established risk factor for tuberculosis (TB), active TB progression and reactivation, poor treatment outcomes and delayed pathogen clearance [1]. Further, T2DM is linked to a threefold increase in the likelihood of active tuberculosis in medium- and low-income countries [2]. The immunological influence of T2DM on tuberculosis has been a topic of discussion, with studies demonstrating that both innate and adaptive immunity are compromised, including elevated pro-inflammatory cytokine responses, increased and impaired neutrophil recruitment, altered monocyte function, and impaired T-cell function, all of which led to impaired immune function, delayed bacterial clearance, and disease progression [3]. These alterations could be attributed to metabolic dysfunction and hyperglycemia in T2DM, which influence the immune cell activity, implying a clear metabolic and immunological interplay [4].

Systemic inflammation is a well-established feature of tuberculosis and type 2 diabetes comorbidity, and prolonged inflammation has been shown to damage lungs with more severe symptoms [5]. Acute-phase proteins (APPs) such as C-Reactive Protein (CRP), Serum Amyloid A1 (SAA1), Haptoglobin, and α2-Macroglobulin (α2M) are regarded as baseline predictors of tuberculosis treatment failure, implying that their potential levels can signal disease severity and serve as possible biomarkers for treatment purposes [5]. Their levels in TB-T2DM patients have yet to be evaluated, as persistent hyperglycemia and metabolic dysfunction may enhance APP synthesis, leading to chronic low-grade inflammation and altered immune signaling.

Lung-associated markers may potentially serve as signs of ongoing lung inflammation. These are mostly produced in the lungs, and their systemic spread can aid in determining the condition of inflammation in the lungs [6]. Markers such as Indoleamine 2,3-dioxygenase (IDO), Chitinase-3-like protein 1 (CHI3L1), S100 Calcium-binding Protein A8 (S100A8), S100 Calcium-binding Protein A9 (S100A9) and granulysin are reported to be elevated in tuberculosis and are associated with dysregulated immune responses, including altered neutrophil recruitment, chronic inflammation, and tissue remodeling, which may contribute to impaired host immunity and persistent infection [7,8,9].

Forming another class of immunoregulatory cytokines are the TNF superfamily ligands (TNFSF-Ligands). These proteins play a major role in controlling infection through immune activation and granuloma formation, such as apoptosis of infected cells, maturation of dendritic cells, activation of macrophages, chemotaxis of T cells and secretion of IFN-γ [10]. Unlike conventional cytokines, TNFSF ligands provides insight into the immune regulatory architecture, reflecting upon cell communication and signaling [11]. Excessive or insufficient production of these cytokines can lead to granuloma disorganization, tissue damage, and exacerbation of inflammation, contributing to TB pathology [12]. Quantifying the circulating levels of this particular family (4-1BBL (TNFSF9), APRIL (TNFSF13), BAFF (TNFSF13B), CD30L (TNFSF8), CD40L (TNFSF5), EDA-1 (TNFSF27), Fas-L (TNFSF6), GITRL (TNFSF18), LIGHT (TNFSF14), RANKL (TNFSF11), NGF- β, TNF-β (TNFSF1), TRAIL (TNFSF10) and TWEAK (TNFSF12)) in TB-T2DM patients can provide information on how persistent metabolic inflammation may alter TNF-mediated signaling and thereby impair effective immune responses and contribute to disease severity.

Beyond biological and immunological factors, psychosocial and socioeconomic circumstances may also influence TB treatment adherence and outcomes, particularly among socially vulnerable populations. Recent evidence highlights the importance of a multidisciplinary approach in improving TB treatment outcomes in these populations [13].

Despite ongoing investigations into inflammatory mediators and their implications for tuberculosis pathology, the combined assessment of acute-phase proteins, lung-specific markers, and TNF family ligands and receptors in TB-T2DM is limited. A thorough understanding of these interrelated networks is required for developing reliable biomarkers of disease severity and optimizing clinical treatment strategies [14]. This study is aimed at understanding the immune regulatory pathways of TNF superfamily ligands associated with TB-T2DM by examining acute-phase proteins, lung-associated markers, and TNF family ligands. This integrated approach provides insight into the overall understanding of the inflammatory niche and its communication with TNF ligand signaling in comorbid individuals and has the potential to provide insights into immune dysregulation mechanisms.

## 2. Materials and Methods

### 2.1. Ethics Statement

The Institutional Ethics Committee of the ICMR-National Institute for Research in Tuberculosis (IEC no. 231/NIRT-IEC/2024) and Institutional Review Board of Saint Louis University (STUDY00000235) granted ethical approval for the study, which was in compliance with the Declaration of Helsinki’s ethical guidelines. All subjects provided written informed consent and granted their permission before enrolment.

### 2.2. Study Cohort

This study included a total of 176 participants recruited at baseline. Participants were categorized into four groups: tuberculosis with type-2 diabetes mellitus (TB-T2DM, *n* = 44), tuberculosis only (TB, *n* = 44), diabetes mellitus only (T2DM, *n* = 44), and healthy controls (HC, *n* = 44). Adults aged ≥18 to 75 years were included in the study (Figure 1). However, the multiplex immunoassay was performed for 22 samples from each cohort due to feasibility constraints. TB–T2DM and TB samples for this study were retrospectively obtained from the Central Biorepository at the ICMR–National Institute for Research in Tuberculosis (NIRT), Chennai, India. Control samples were retrospectively sourced from the RePORT Phase I parent protocol of the Effect of Diabetes on Tuberculosis Severity (EDOTS) study conducted at NIRT, Chennai. Controls were selected based on age, sex, BMI, T2DM status, and LTBI status. Pulmonary tuberculosis was diagnosed based on sputum smear microscopy and culture positivity. Chest radiographs were used to assess the presence of cavitary disease and to classify lung involvement as unilateral or bilateral. Bacterial burden was determined using sputum smear grading and categorized as 1+, 2+, or 3+. At the time of enrolment, all active TB participants had no prior history of tuberculosis or anti-tuberculosis treatment. Type 2 diabetes mellitus was diagnosed using a 75 g oral glucose tolerance test (OGTT, 2 h) or glycated hemoglobin (HbA1c) levels as HbA1c ≥ 6.5%, according to standard criteria. However, other cohorts such as TB-T2DM, TB only and healthy controls were measured for glycemic status by glycated hemoglobin (HbA1c) levels only. Pregnant or lactating women and HIV-positive individuals were excluded from the cohort (Table 1).

### 2.3. Acute-Phase Proteins and Lung-Associated Markers

The study quantified a proposed panel of acute-phase proteins (APPs) and lung-associated inflammatory markers, including C-Reactive protein (CRP), haptoglobin, Serum Amyloid A1 (SAA1), α-2-macroglobulin (α2M), Indoleamine 2,3- dioxygenase (IDO), Chitinase 3-like 1 (CHI3L1), S100A8, S100A9, and Granulysin. Each biomarker was measured independently using commercially available DuoSet™ ELISA kits (R&D Systems, Minneapolis, MN, USA). All assays were conducted in accordance with the manufacturer’s protocols, utilizing the DuoSet™ Ancillary Reagent Kit.

### 2.4. TNF Superfamily Ligands

Plasma levels of TNF superfamily (ligands) cytokines (4-1BBL, APRIL, BAFF, CD30L, CD40L, EDA-1, Fas-L, GITRL, LIGHT, NGF-β, RANKL, TNF-α, TNF-β, TRAIL and TWEAK) were quantified using a bead-based multiplex immunoassay based on Luminex xMAP technology with a custom 15-plex multiplex ELISA kit (ProcartaPlex™ Human, NHP, and Canine Mix & Match Panels (Invitrogen, Thermo Fisher Scientific, Waltham, MA, USA)). Data acquisition was performed using MAGPIX™ platform, and concentrations were determined using standard curve-based analysis.

### 2.5. Statistical Analysis

#### 2.5.1. Data Distribution and Group Comparisons

Statistical evaluations were performed using Graph Pad Prism software (version 11). As most variables showed non-normal distribution, non-parametric statistical tests were used. Kruskal Wallis test (*p* < 0.05—significant) was performed to assess differences among the four primary study groups TB-T2DM, TB, T2DM and healthy controls, followed by Dunn’s multiple comparisons test for pairwise comparisons. Adjusted *p* values were considered statistically significant at *p* < 0.05. Additionally, Spearman’s rank correlation analysis was conducted to examine associations between HbA1c levels and analyte concentrations across different groups. This analysis aimed to determine whether elevations in acute-phase proteins observed in the TB-T2DM group were attributable to diabetes status and was carried out using JMP Statistical Software (SAS Institute) (version JMP 17.0.0). Heat maps were generated using pHeatmap in R software (version 2026.04.0).

#### 2.5.2. Pathway Enrichment Analysis

A protein–protein interaction (PPI) network of differentially expressed cytokines was constructed using the STRING database (version 11), applying a medium confidence interaction score (0.7). The network was based on known and predicted protein–protein associations, including inhibition, binding, activation, catalysis, transcriptional regulation, and post-translational modifications. Pathway enrichment analysis was performed using Enrichr database version 12. Overrepresented biological processes were identified using a hypergeometric test. Multiple testing correction was applied using the Benjamini–Hochberg method, and pathways with a false discovery rate (FDR) <0.01 were considered statistically significant. Enrichment results included KEGG pathways, Gene Ontology (biological processes) and Reactome pathways.

## 3. Results

### 3.1. Differential Expression of Acute-Phase Proteins and Lung Markers

Circulating levels of acute-phase proteins and lung-associated markers were evaluated across the four study groups (*n* = 44). Significant alterations were observed in multiple inflammatory markers, including C-reactive protein (CRP), α2-macroglobulin (A2M), serum amyloid A1 (SAA1), haptoglobin, indoleamine 2,3-dioxygenase (IDO), chitinase-3-like protein 1 (CHI3L1), S100A8, S100A9, and granulysin.

Among the groups, individuals with TB- T2DM demonstrated the highest inflammatory burden, with markedly increased levels of several acute-phase proteins and certain lung markers when compared with healthy controls. However, elevated significance was observed compared to TB- or T2DM-alone individuals, indicating an amplified inflammatory state during the comorbidity. Particularly, robust upregulation in TB-T2DM individuals was well evident in CRP, α2M, SAA1, Haptoglobin, S100A8, S100A9, and CH3IL1 (Figure 2).

### 3.2. Altered TNF-Superfamily Ligand Profile in TB-T2DM Individuals

The circulating levels of TNF superfamily ligands were quantified to understand the alteration in TNF-superfamily ligand signaling (*n* = 22). Significant differences were observed for BAFF, CD30L, CD40L, EDA-1, APRIL, GITRL, LIGHT, and NGF-β across the study groups. The TB-T2DM group showed substantial dysregulation of several TNF ligands compared with healthy controls, TB alone or T2DM individuals. Ligand molecules associated with immune activation and apoptosis, including APRIL, BAFF, GITRL TRAIL, CD40L and LIGHT, exhibited marked elevation in TB-T2DM individuals. These findings indicate enhanced TNF-mediated inflammatory and immune signaling in individuals with concurrent TB and T2DM (Figure 3).

### 3.3. Association Between TNF-Superfamily Ligands and Differentially Expressed Biomarkers

A correlation analysis was performed between the TNF-superfamily ligands (*n* = 22) and the differentially expressed APPs and lung markers (*n* = 44). The network illustrates the correlation between the biomarkers representing positive correlations with red edges, negative correlations with blue edges and the strength of association indicated by the thickness of the edges. The plot shows that among the acute-phase proteins, SAA1 exhibited strong positive correlations with many TNF-superfamily ligands, especially TRAIL and BAFF, suggesting a possible involvement in inflammatory signaling pathways. Haptoglobin and CRP also demonstrated moderate positive interactions with immune-related ligands. In contrast, certain markers such as A2M and S100A9 displayed negative correlations with selected TNF ligands, indicating potential regulatory or opposing inflammatory responses. The network suggests there is a complex interplay between inflammatory biomarkers and TNF-superfamily ligands, suggesting coordinated regulation of immune and inflammatory responses in TB-T2DM patients (Figure 4).

### 3.4. Correlation Analysis Demonstrating Significant Positive Associations of the Proposed Biomarkers with HbA1C Levels in TB-T2DM Patients:

A nonparametric Spearman’s rank correlation analysis was performed among HbA1c values and acute-phase proteins, lung markers and TNF-superfamily ligands to evaluate the direction of their associations. The correlation coefficients (Spearman’s ρ) and corresponding *p*-values indicate the strength and significance of the association between HbA1c and each biomarker. Among the biomarkers analyzed, TRAIL exhibited the strongest positive correlation (ρ = 0.58), followed by GITRL (ρ = 0.56), BAFF (ρ = 0.51), and LIGHT (ρ = 0.50), indicating enhanced inflammatory and immune activation with increasing HbA1c levels. Moderate positive correlations were also observed for CD40L (ρ = 0.41), APRIL (ρ = 0.37), SAA1 (ρ = 0.31) and S100A8 (ρ = 0.374). All had significant correlation with *p* < 0.05. Hierarchical clustering analysis further demonstrated close grouping of TNF-superfamily ligands and inflammatory mediators, indicating coordinated regulation of immune and inflammatory pathways. This suggests a strong relationship between glycemic status and inflammatory immune responses in the studied condition (Figure 5).

### 3.5. Protein–Protein Interaction Network and Functional Enrichment Analysis of Significant Inflammatory and TNF-Superfamily Biomarkers

Protein–protein interaction (PPI) networks and functional enrichment analysis were performed using STRING with a confidence level of *p* > 0.7. The interaction network revealed TNF as a central hub protein with extensive connectivity to multiple inflammatory mediators, including TNFSF members like FASLG and CD40LG and inflammatory markers like CRP, SAA1, and S100A8/S100A9, indicating its major regulatory role in immune and inflammatory signaling. Strong interactions were also observed among TNFSF proteins and acute-phase markers, suggesting coordinated immune activation and cytokine-mediated responses. Gene Ontology (GO) biological process analysis demonstrated the network was enriched in pathways related to regulation of B-cell proliferation, endothelial cell migration, positive regulation of NF-κB transcription factor activity, positive regulation of cell proliferation, and T-cell activation. Cellular component analysis showed significant enrichment in vesicles, ficolin-1-rich granules, cytoskeleton, secretory granules, extracellular matrix and cytoplasmic vesicle lumen, suggesting active immune secretion and extracellular signaling mechanisms. Molecular function analysis showed enrichment primarily in zinc ion binding, indicating strong immune receptor-mediated signaling activity. KEGG pathway enrichment analysis further identified significant involvement of necroptosis, apoptosis, natural killer cell-mediated cytotoxicity, FOXO signaling pathway, prolactin signaling pathway, IL-17 signaling pathway, NF-κB signaling pathway and cytokine–cytokine receptor interaction pathways. These findings collectively suggest that the identified biomarkers are strongly associated with inflammatory signaling, immune regulation, apoptosis and cytokine-mediated cellular responses in TD-T2DM groups (Figure 6).

## 4. Discussion

Tuberculosis (TB) and type 2 diabetes mellitus (T2DM) comorbidity is a distinct immunometabolic condition, with T2DM elevating TB progression risk by two- to three-fold. It is well evident that their coexistence is characterized by persistent inflammation, impaired immune regulation and altered host immune responses. Previous studies from our laboratory have shown that TB-T2DM individuals demonstrate exaggerated inflammatory responses even during anti-TB treatment and also demonstrate altered monocyte associated TNF-superfamily receptor signaling compared to other groups [15]. TNF-superfamily ligands signaling has a significant role in T-cell and B-cell regulation, macrophage activation and granuloma maintenance in TB. Further, several studies reported earlier focused only on isolated inflammatory mediators; hence, this approach of evaluating TNF-superfamily ligands together with acute-phase proteins and lung markers and understanding their association provides a more comprehensive understanding of the interconnected inflammatory and immune regulatory networks involved in TB-T2DM comorbidity.

In this study, significantly elevated levels of acute-phase proteins and lung-associated inflammatory markers were observed in TB-T2DM individuals compared to TB-alone, T2DM-alone and healthy controls. Particularly, CRP, α2M, SAA1, S100A8, S100A9, CHI3L1 and haptoglobin were elevated in the TB-T2DM cohort, and could be associated with the presence of an amplified systemic inflammatory state. Acute-phase proteins are induced in response to inflammatory cytokines such as IL-6, IL-1β and TNF-α and are closely associated with chronic inflammatory conditions and pulmonary tissue injury. Increased CRP and SAA1 levels have been previously reported to be linked with active pulmonary TB and poor glycemic control [16]. Elevated CRP levels in the TB-T2DM cohort are consistent with the studies of NP Kumar et al. 2019 [17], which demonstrated significantly higher CRP levels in TB-T2DM individuals compared to TB-alone, with levels remaining elevated throughout the anti-tuberculosis treatment. This persistent CRP elevation, with positive correlations between CRP levels and HbA1c, reflects the association between glycemic control and inflammatory burden [17,18,19]. Similarly, the S100 family proteins such as S100A8 and S100A9 act as crucial mediators of inflammatory amplification and neutrophil recruitment during active TB disease. Their heightened expression tends to be associated with increased myeloid activation and pulmonary pathology. While our study examined S100A8 and S100A9, the study by N.P. Kumar et al., 2019 [20] demonstrated elevated S100A12 in TB-T2DM patients, indicating broader S100 family dysregulation. Higher S100A12 levels correlating with disease extent in patients with bilateral and cavitary disease suggest these proteins reflect pulmonary pathology severity. In T2DM, these proteins are associated with chronic inflammatory stress and endothelial dysfunction [9,20]. Increased CHI3L1 levels further support our findings, suggesting inflammatory lung remodeling and macrophage activation [21]. Collectively, the findings from our study suggest that hyperglycemia may be associated with an exaggerated inflammatory response during TB infection, which can contribute to a prolonged inflammatory state in TB-T2DM individuals.

TNF-superfamily ligands represent a functionally significant group of immune mediators involved in macrophage activation, granuloma maintenance, apoptosis, T-cell and B-cell regulation, and cytokine signaling. These mechanisms are essential for host immune responses against Mycobacterium infection [22]. In TB-T2DM comorbidity, chronic hyperglycemia and metabolic stress tend to create an imbalance which may be associated with exaggerated inflammation, impaired immune regulation, delayed bacterial clearance and pulmonary tissue damage [3,11]. Hence, profiling these TNF-superfamily ligands may help us to understand the associated underlying immune-metabolic dysregulation in TB-T2DM comorbidity beyond the inflammatory signatures. Circulating levels of TNF ligands, such as BAFF, APRIL, CD40L, GITRL, LIGHT, TRAIL, and EDA-1, exhibited a distinct immune profile across the study groups, showing significant alterations in TB-T2DM individuals. Increased levels of CD40L observed in TB-T2DM individuals may explain the crosstalk of inflammatory responses between antigen-presenting cells and T lymphocytes. CD40-CD40L signaling plays an essential role in macrophage activation and cytokine production during *M.tb* infection [23]. Increased expression of BAFF and APRIL may indicate persistent adaptive immune activation and dysregulated B-cell survival during chronic inflammation [24]. Studies by Kai-zhong Liu et al. 2012 showed that BAFF and APRIL are upregulated in tuberculosis patients, depicting their biomarker potential [25]. However, studies of these ligands in TB-T2DM individuals are not reported. Altered expression of TRAIL suggests a dysregulated apoptosis-mediated immune mechanism, which is considered as an essential host defense mechanism limiting the intracellular survival of *M.tb*. Alterations in this mechanism may be associated with tissue destruction and disease progression [22,26,27]. Increased levels of GITRL and LIGHT may indicate altered T-cell activation and inflammatory signaling, while reduced EDA-1 levels may reflect changes in pathways involved in immune regulation and tissue homeostasis. These alterations, together with the broader immune balance reported in T2DM (Minette Lagman et al., 2015) [28], may reflect the complex immune metabolic environment associated with the disease. Though GITR-related signaling has been associated with immune cell function and glucose homeostasis, Galle-Treger et al. (2019) [29] investigated GITR rather than GITRL and did not include TB. Further investigation of these ligands is required [28,29]. Together, these findings suggest that an altered TNF-superfamily ligand profile may reflect persistent inflammation, prolonged inflammatory amplification and altered immune regulation associated with chronic hyperglycemia.

Previous studies have demonstrated the distinct inflammatory cytokine signatures in TB-T2DM individuals, while the coordinated relationship between acute-phase proteins, lung-associated inflammatory markers and TNF-superfamily ligand signaling is not completely understood. In a correlation plot between the TNF-superfamily ligands and significant acute-phase proteins, lung markers reveal positive associations, demonstrating the presence of interconnected inflammatory networks in TB-T2DM individuals. These connections between TNF ligands and markers such as CRP, SAA1, A2M, S100A9 and Haptoglobin may indicate coordinated regulation between innate immune mechanisms, systemic inflammatory responses and TNF-mediated signaling pathways. These balanced inflammatory responses, which are regulated through receptor- and cytokine-mediated communications, may be altered in the context of persistent hyperglycemia through various mechanisms, including prolonged oxidative stress, AGE formation and persistent NF-κB activation [30,31]. This sustained signaling and coordinated elevation of TNF ligands, APPs and lung markers suggests that TNF-mediated immune activation may contribute to persistent inflammatory responses in TB-T2DM individuals. Similar inflammatory mechanism network interactions have been reported in other chronic and metabolic diseases where prolonged inflammation leads to dysregulated immune mechanisms [15,32].

Several acute-phase proteins, lung-associated markers and TNF-superfamily ligands demonstrated significant positive correlations with HbA1C, suggesting the interconnection between the dysregulated immune networks and prolonged inflammatory responses in chronic hyperglycemic individuals with TB. Prolonged increases in glycemic levels may promote generation of advanced glycation end-products (AGEs), reactive oxygen species (ROS) and inflammatory cytokines, contributing to immune dysregulation and defective host immune responses [33]. Previous studies have reported that poor glycemic control in TB-affected individuals is often associated with elevated inflammatory cytokines and altered immune responses [33,34,35]. In addition, studies in individuals with diabetes have demonstrated associations between higher HbA1c levels and increased inflammatory markers such as CRP, IL-6 and TNF-α. However, longitudinal evidence suggests a close relationship between inflammation and glycemic dysregulation [36,37]. Further, experimental evidence demonstrates that hyperglycemia may promote oxidative stress and inflammatory responses, including increased production of ROS and pro-inflammatory cytokines, including IL-6 and TNF-α [38]. Previous studies have also reported associations between altered glycemic status and poorer clinical outcomes in other populations [39]. In our study, positive associations observed between APPs, lung markers and TNF ligands may indicate that there is a relationship between glycemic status and inflammatory immune response, and these findings may indicate an association between glycemic status and TNF-superfamily-mediated signaling during TB infections. Prolonged glycemic imbalance has also been associated with impaired macrophage function, antigen presentation and delayed bacterial clearance, contributing to poor clinical outcomes, frequently observed in TB-T2DM individuals [3]. As this was an observational study, these associations cannot be attributed to the relationship with inflammation and adverse clinical outcomes.

The protein–protein interaction and pathway enrichment analyses revealed an interconnectivity between TNF-superfamily ligands, acute-phase proteins and lung-associated markers, suggesting a possible communication between TNF ligand signaling and associated inflammatory networks. Several molecules that appeared as central hubs were associated with immune activation, cytokine signaling, apoptosis and inflammatory amplification. KEGG pathway enrichment analysis demonstrated significant enrichment of cytokine–cytokine receptor interaction, NF-κB signaling, IL-17 signaling, apoptosis and necroptosis pathways. NF-κB signaling is a crucial central regulator of the inflammatory transcriptional network, involved in the chronic metabolic inflammation [31]. Additionally, IL-17 signaling has been associated with pulmonary tissue pathology and neutrophilic inflammation during TB infection [40]. Interestingly, the enrichment of apoptosis and necroptosis pathways suggests that dysregulated cell death mechanisms with impairment in the apoptosis pathway may be associated with altered cell death responses during *M.tb* pathogenesis. Sustained and prolonged activation of these pathways may contribute to exaggerated inflammatory responses, as observed in comorbid individuals [26,27,31,40]. Gene ontology analysis revealed enrichment of T-cell activation, B-cell proliferation and positive regulation of NF-κB signaling, further substantiating our findings. Therefore, the pathway enrichment findings suggest an association of TNF-superfamily signaling with inflammatory and immune regulatory pathways in TB-T2DM; however, these findings require further functional validation. However, our study has a few limitations. Initially, this was a cross-sectional study evaluating the hyperinflammatory responses and immune dysregulation in individuals at specific time points, and further follow-up studies are required to draw specific conclusions. Although the study groups were equally distributed, the limited sample size and the use of a subset of 22 participants per group for multiplex immunoassays due to kit availability and feasibility constraints may limit the generalizability of the findings. Hence, the findings from this study should be interpreted with consideration and validated in larger cohorts. Similarly, no adjustment for multiple comparisons across the groups were applied to the exploratory correlation analysis, and hence, the findings should be cautiously interpreted. Also, this study was primarily an observational and descriptive study, and hence, further functional studies are required to reveal the mechanistic underpinnings of how T2DM impairs the inflammatory and TNF signaling axis in TB individuals.

In conclusion, our study underlines the significant contribution of TNF-superfamily ligand-mediated inflammatory signaling in TB-T2DM comorbidity. Combined elevation of acute-phase proteins, lung-associated inflammatory markers and TNF ligands reveals that hyperglycemia may contribute to amplified immune dysregulation during TB infection. Correlation and network analyses further demonstrated interconnected inflammatory signaling involving apoptosis, cytokine–cytokine signaling and NF-κB signaling. These findings indicate that persistent hyperglycemia can alter the TNF-mediated immune communication, contributing to exaggerated inflammation, pulmonary tissue injury and impaired immune regulation in TB-T2DM individuals. The identified TNF ligands and associated inflammatory markers may thus represent potential inflammatory signatures of disease severity and could provide future insights into further pathway-related investigations with clinical and biological relevance in TB-T2DM comorbidity.

## Figures and Tables

**Figure 1:**
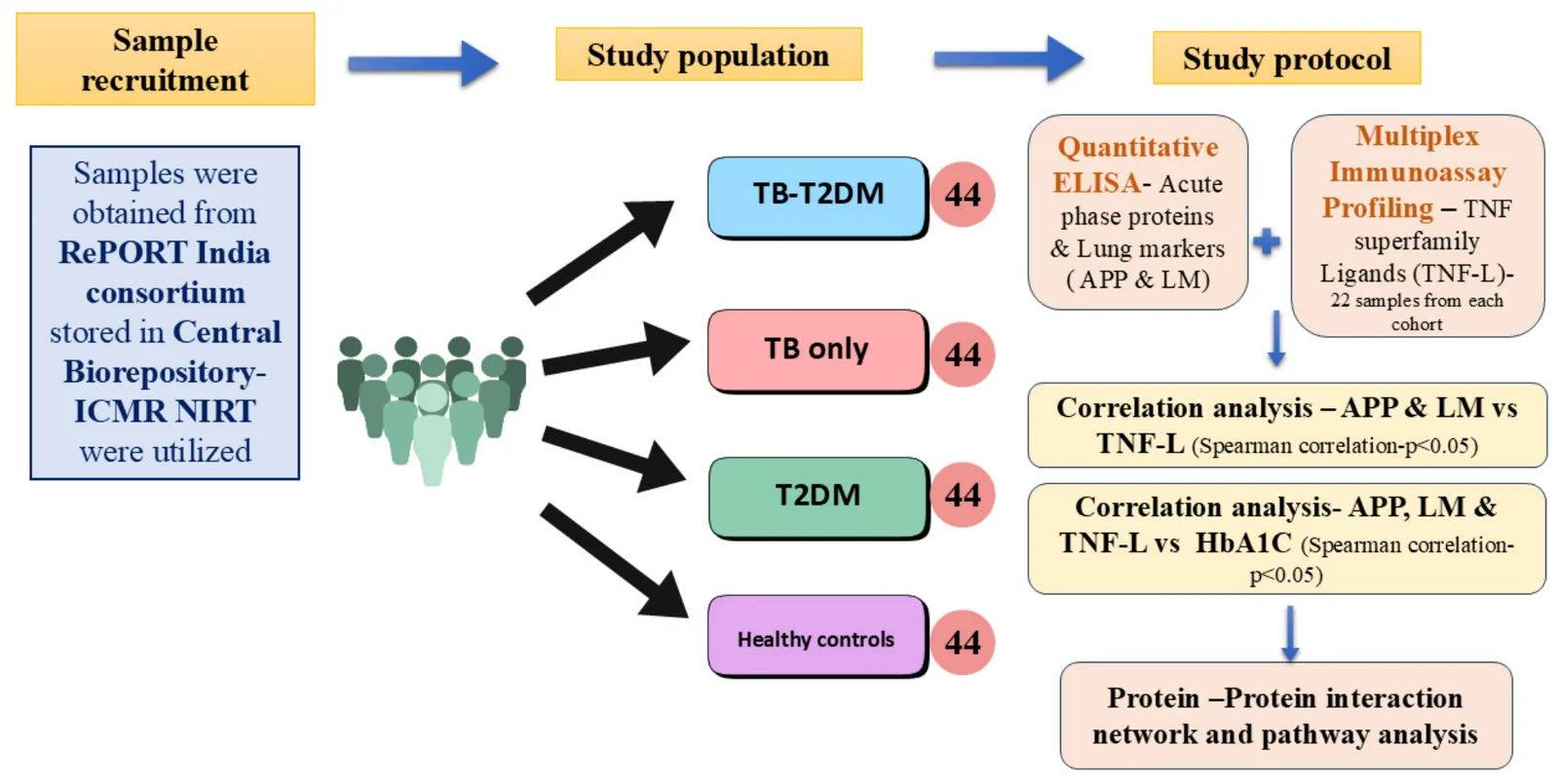
Study design and workflow. The figure illustrates schematic representation of the study design and experimental workflow. Plasma samples were categorized into four groups: Group 1—TB-T2DM (pulmonary TB patients with T2DM—44), Group 2—T2DM (T2DM without active or latent TB—44), Group 3—TB (pulmonary TB patients without T2DM—44) and Group 4—healthy controls (without active or latent TB or T2DM—44). Samples were obtained from RePORT India consortium and stored in Central biorepository; of ICMR-NIRT. These samples were utilized for this study. Multiplex immunoassay profiling was performed to quantify circulating TNF superfamily ligands (TNF-L), while quantitative ELISA was performed for acute-phase proteins (APPs) and lung-associated markers (LMs). However, multiplex immunoassay was performed for only 22 samples from each cohort (TB = 22, TB–T2DM = 22, T2DM-only = 22 and healthy controls = 22). Further, correlation analysis between APPs and LMs, correlation analysis between APPs, LMs and HbA1c, and protein–protein interaction/pathway enrichment analyses were subsequently performed to evaluate the association between inflammatory markers, TNF ligands and hyperglycemia.

**Figure 2:**
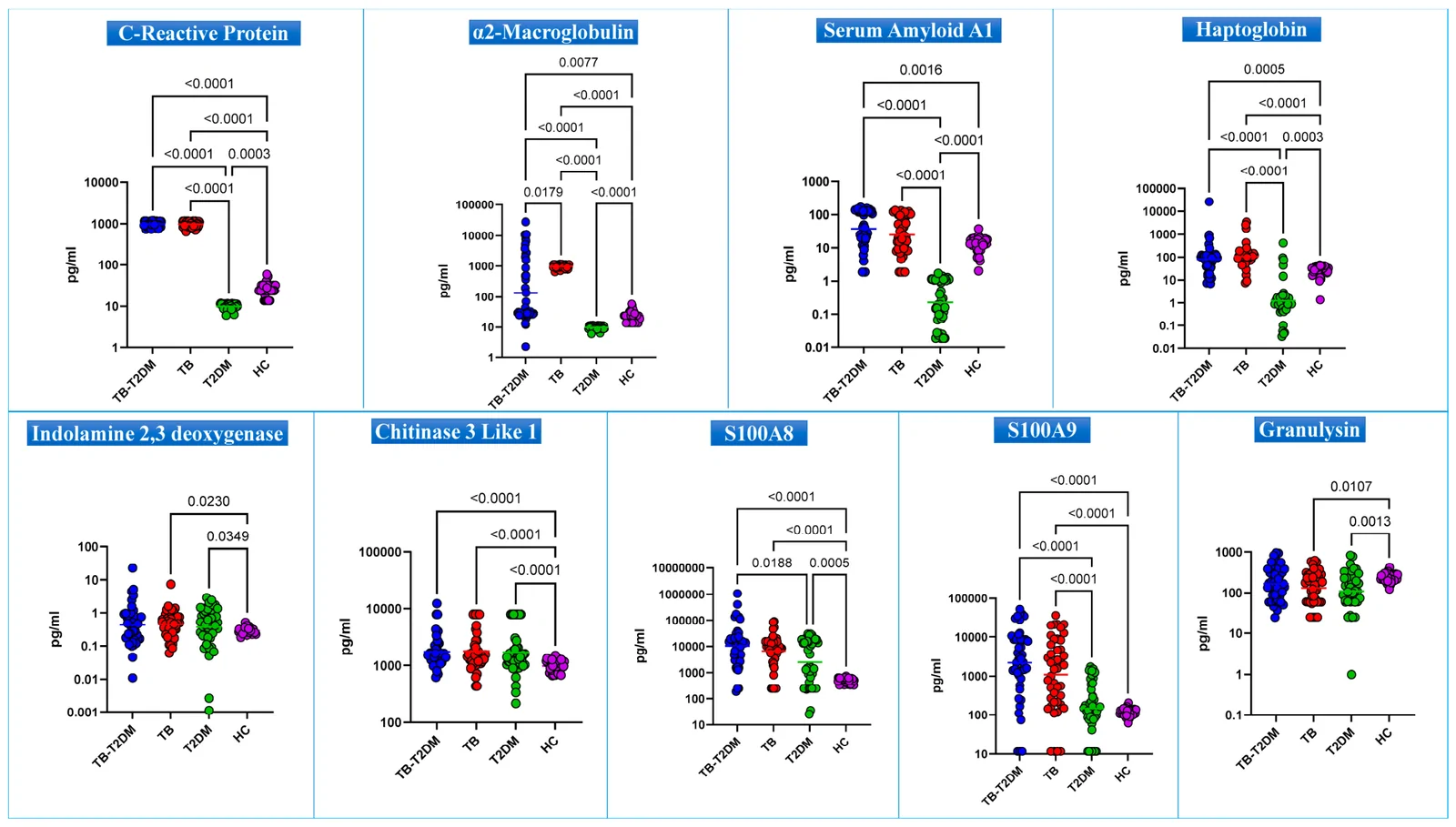
Acute-phase protein and lung-associated marker expression profiles across the TB-T2DM, TB, T2DM and healthy control study groups. This figure illustrates the panel of acute-phase proteins (APPs) and lung-associated markers (LMs) analyzed in the study (*n* = 44). Scatter plots depict concentrations (pg/mL) of C-reactive protein (CRP), α2 macroglobulin (A2M), serum amyloid A1 (SAA1), haptoglobin, indoleamine2-3, dioxygenase (IDO), chitinase-3-like protein 1 (CHI3L1), S100A8, S100A9 and granulysin. Each dot represents an individual participant, and horizontal bars represent the median values. Significant group-wise differences were observed in individuals with TB-T2DM compared with TB, T2DM, and healthy controls (*p* < 0.05). Elevated circulating levels of CRP, α2M, SAA1, Haptoglobin, S100A8, S100A9, and CH3IL1 were predominantly observed in TB-T2DM individuals (*p* < 0.005).

**Figure 3:**
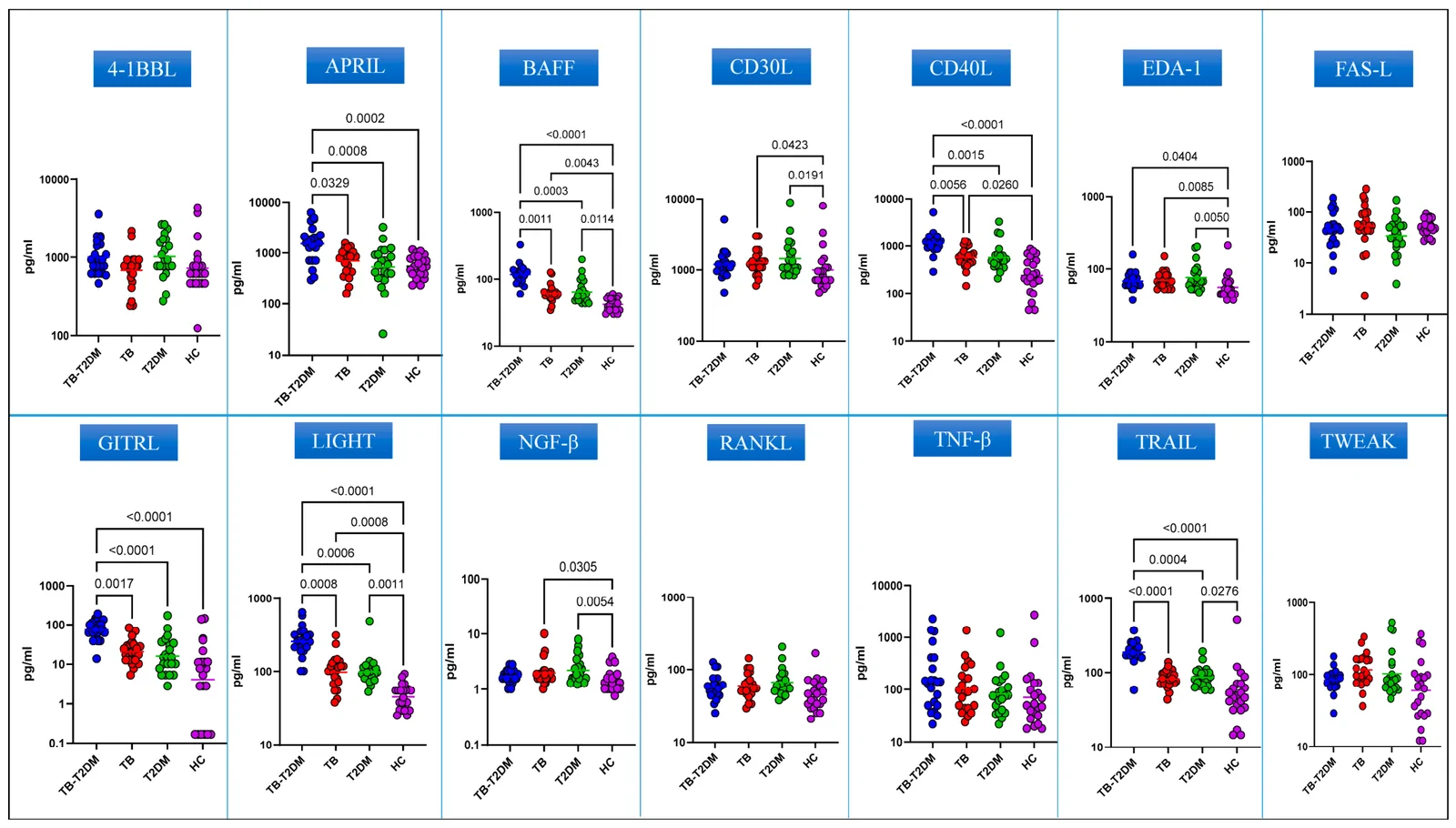
Circulating TNF-superfamily ligand expression profile across the TB-T2DM, TB, T2DM and healthy control study groups. The figure illustrates the plasma levels of soluble TNF-superfamily ligands measured in TB-T2DM, TB, T2DM and healthy controls (HC) (*n* = 22). Scatter plots depict concentrations (pg/mL) of 4-1BBL, APRIL, BAFF, CD30L, CD40L, EDA-1, Fas-L, GITRL, LIGHT, NGF-β, RANKL, TNF-α, TNF-β, TRAIL and TWEAK. Each dot represents an individual participant, and horizontal bars represent the median values. Significant group-wise differences were observed in individuals with TB-T2DM compared with TB, T2DM, and healthy controls (*p* < 0.05). Increased circulating levels of BAFF, APRIL, GITRL, LIGHT, CD40L, TRAIL and LIGHT and decreased levels of EDA-1 were predominantly observed in TB-T2DM individuals compared to other groups.

**Figure 4:**
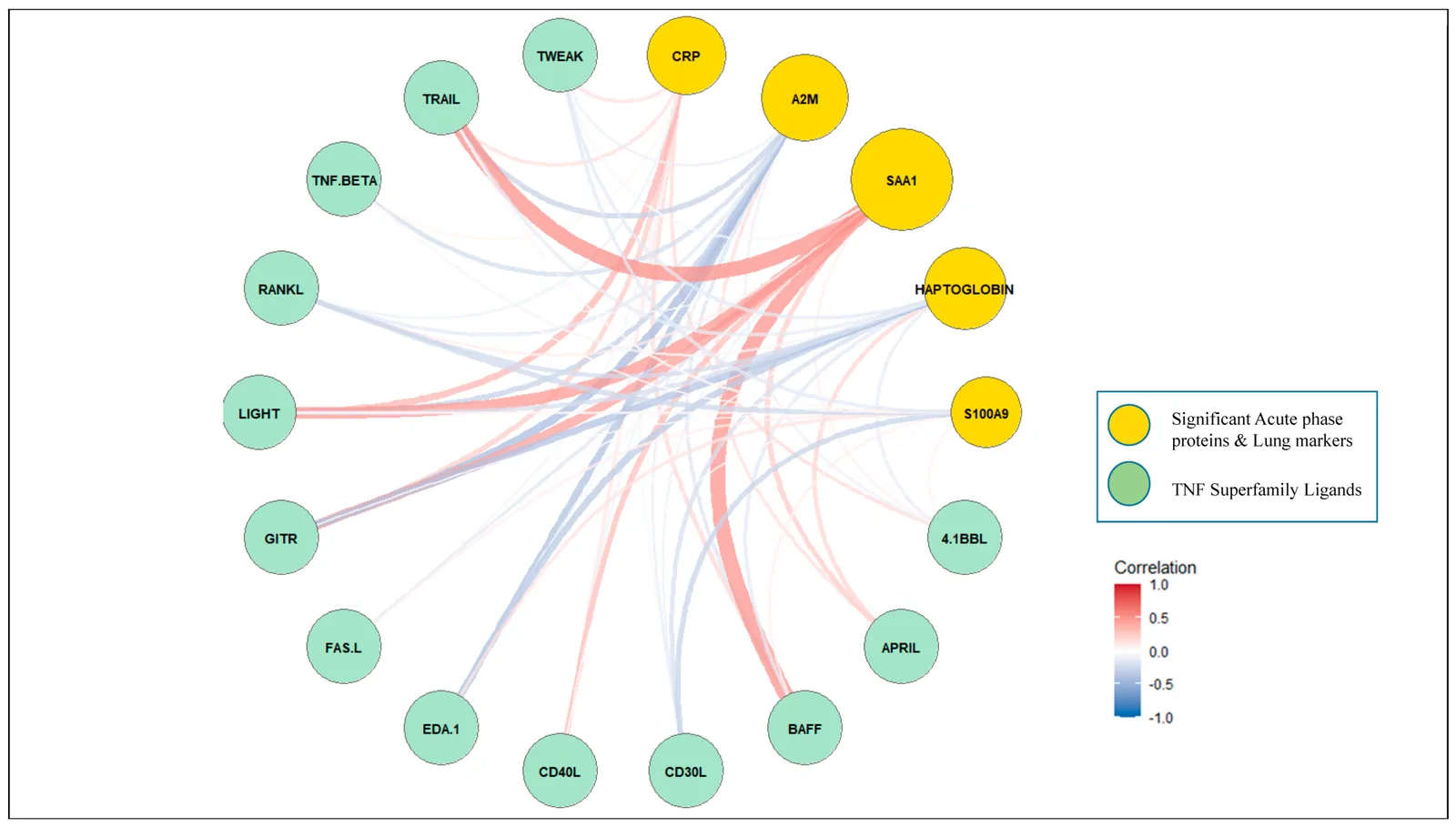
Correlation network analysis between significant acute-phase proteins, lung markers and TNF-superfamily ligands. The figure represents the network plot illustrating the statistically significant Spearman correlations between significantly altered acute-phase proteins (APPs), lung markers (LMs) [*n* = 44] and TNF-superfamily ligands (TNF-L) [*n* = 22] across the four study groups. Yellow nodes represent significant acute-phase proteins and lung markers including CRP, A2M, SAA1, haptoglobin and S100A9, while green nodes represent TNF-superfamily ligands including BAFF, CD30L, CD40L, EDA-1, FAS-L, GITRL, LIGHT, RANKL, TNF-β, TRAIL, TWEAK, APRIL and 4-1BBL. Red connecting edges indicate positive correlations, whereas blue edges represent negative correlations. Edge thickness corresponds to the strength of the correlation coefficient, ranging from −1 to +1. Strong positive correlations were predominantly observed in SAA1, forming a larger yellow node.

**Figure 5:**
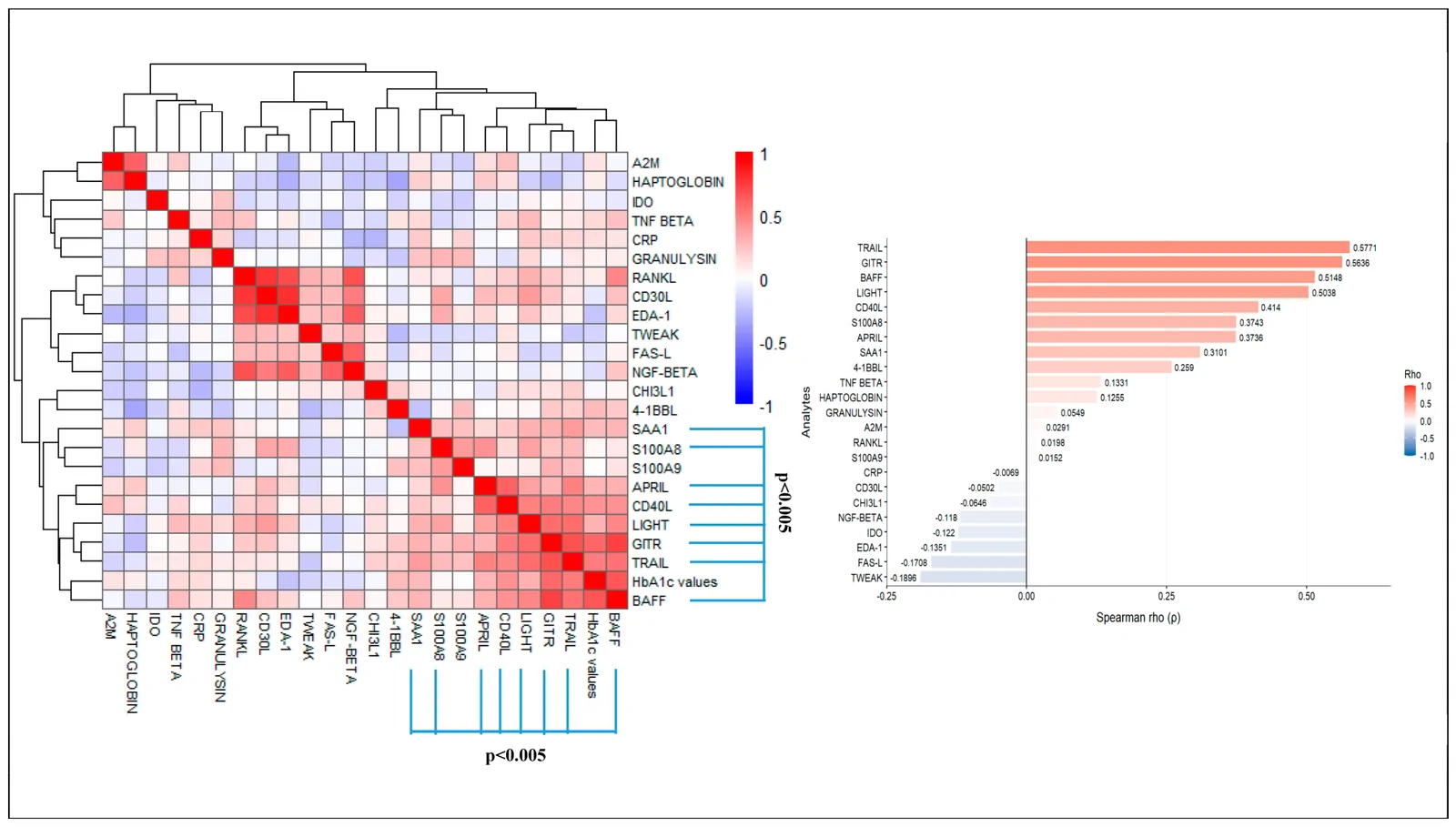
Correlation analysis of acute-phase proteins, lung-associated markers and TNF-superfamily ligands with HbA1c. The figure illustrates the heatmap of Spearman correlation analysis of the associations between acute-phase proteins (APPs) and lung markers (LMs) [*n* = 44], TNF-superfamily ligands (TNF-L) [*n* = 22] and HbA1c values across the study groups. The hierarchical clustering of the heatmap demonstrates positive and negative correlations between the biomarkers, whereas red color represents positive correlations, and blue color represents negative correlations, with correlation coefficients ranging from −1 to +1. Significant positive correlations were observed among BAFF, TRAIL, GITRL, LIGHT, CD40L, APRIL, S100A8 and SAA1 (*p* < 0.05) (left). The rho (ρ) plot represents the strength and direction of correlations between the individual analytes and HbA1c.

**Figure 6:**
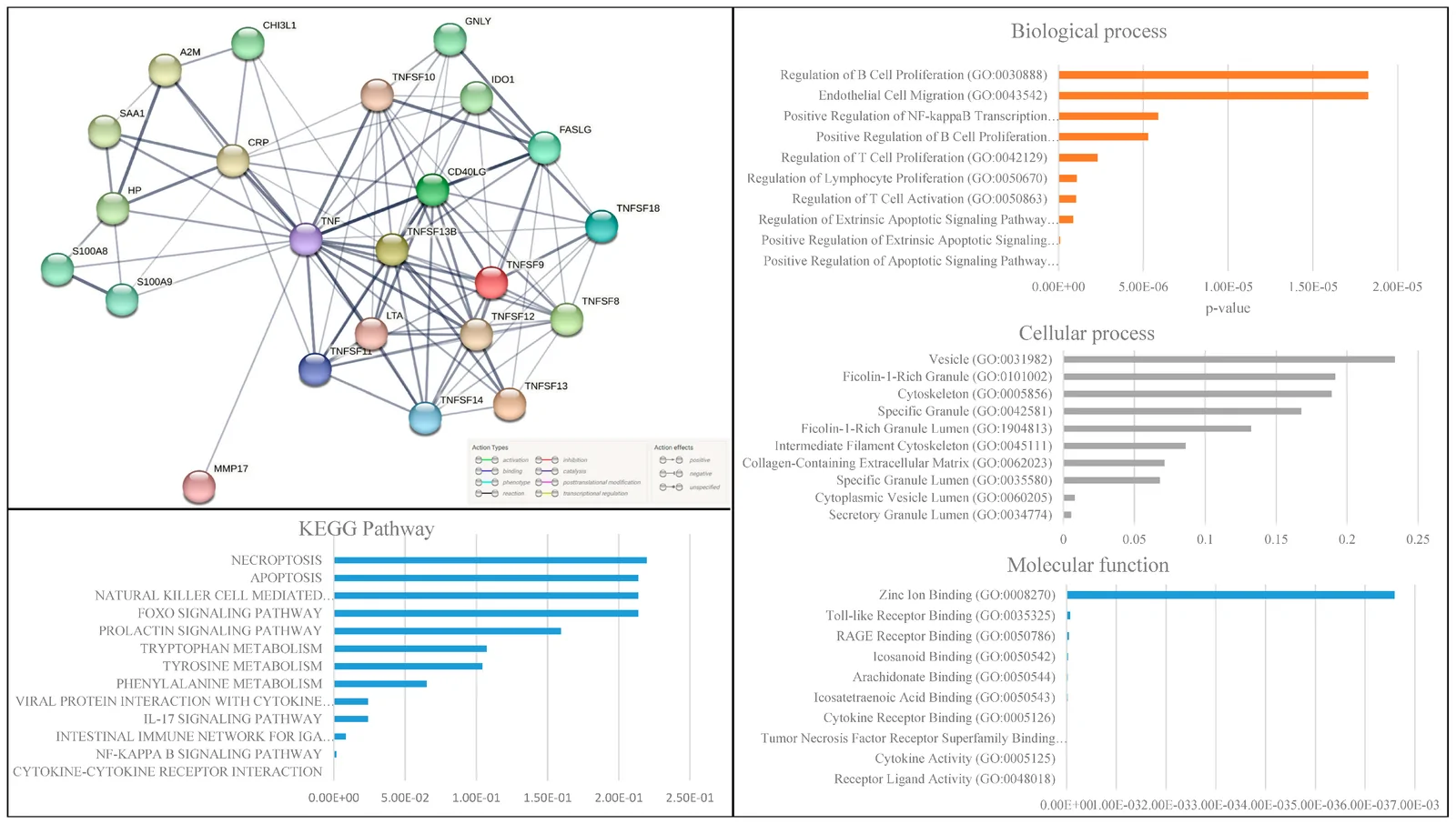
Acute-phase protein, lung marker and TNF-superfamily ligand network and pathway enrichment analysis. Protein–protein interaction (PPI) network and pathway enrichment analysis of significantly altered TNF-superfamily ligands and associated inflammatory markers. KEGG pathway enrichment analysis demonstrated significant enrichment in cytokine–cytokine receptor interaction, NF-κB signaling pathway, IL-17 signaling pathway, and apoptosis, necroptosis and natural-killer cell-mediated cytotoxicity pathways. Gene Ontology (GO) enrichment analysis identified biological processes associated with the regulation of apoptotic signaling, T-cell activation and lymphocyte proliferation. Cellular component analysis revealed enrichment in secretory granule lumen, cytoplasmic vesicle lumen and extracellular matrix-associated compartments, while molecular function analysis highlighted cytokine activity, receptor ligand activity and TNF-superfamily receptor binding.

**Table 1:** Study demographics—the table presents the baseline demographic and clinical characteristics of the study groups, including age, sex distribution, glycemic status, BMI, lifestyle factors, radiological findings, microbiological results, and treatment outcomes.

Parameters	TB-T2DM (*n* = 44)	TB (*n* = 44)	T2DM (*n* = 44)	Healthy Controls (*n* = 44)	*p*-Value
**HbA1c**	10.55 (8–11.97)	5.8 (5.5–6.1)	9.88 (6.95–11.17)	5.59 (5.38–5.82)	<0.00001
**Age (years)**	47.5 (40.75–55.25)	30 (22–38.5)	52 (43.5–57.25)	35 (28–42)	<0.0001
**Sex (M: F)**	29:15	23:21	30:14	22:22	<0.00001
**BMI**	20.15 (18.2–23)	17.35 (15.97–18.95)	26.8 (25.85–28.3)	28.2 (24.8–29.15)	<0.00001
**Smoking (%)**	72.40%	52.20%	NA	NA	0.0475
**Alcoholism (%)**	62.10%	56.50%	NA	NA	0.372
**Cavity present (%)**	24 (54.5%)	34 (77.3%)	NA	NA	0.069
**Chest X-ray score**	53 (20–65.25)	65 (53.75–80)	NA	NA	0.018
**Smear positive (%)**	10 (22.7%)	17 (38.6%)	NA	NA	0.108
**Culture positive (MGIT)**	44 (100%)	43 (97.7%)	NA	NA	0.3145

Demographic continuous variables are presented as median (P25 and P75). Comparisons among the four study groups were performed using the Kruskal–Wallis test, while comparisons between TB-T2DM and TB groups were performed using the Mann–Whitney U test. Categorical variables are presented as *n* (%) and were compared using the chi-square test. *p* < 0.05 was considered statistically significant.

## Data Availability

The original contributions presented in this study are included in the article. Further inquiries can be directed to the corresponding author.
